# Supplementary material for: Normative Modeling of Static and Dynamic Functional Connectivity
Source: bioRxiv. 2026 Apr 6:2026.04.03.716292. Preprint. [Version 1] doi: 10.64898/2026.04.03.716292 (PMC13082073; doi:10.64898/2026.04.03.716292)
Supplement: 1 [file NIHPP2026.04.03.716292V1-supplement-1.pdf]

bioRxiv preprint doi: https://doi.org/10.64898/2026.04.03.716292; this version posted April 6, 2026. The copyright holder for this preprint (which was not certified by peer review) is the author/funder, who has granted bioRxiv a license to display the preprint in perpetuity. It is made available under aCC-BY 4.0 International license.

## 5. Supplementary

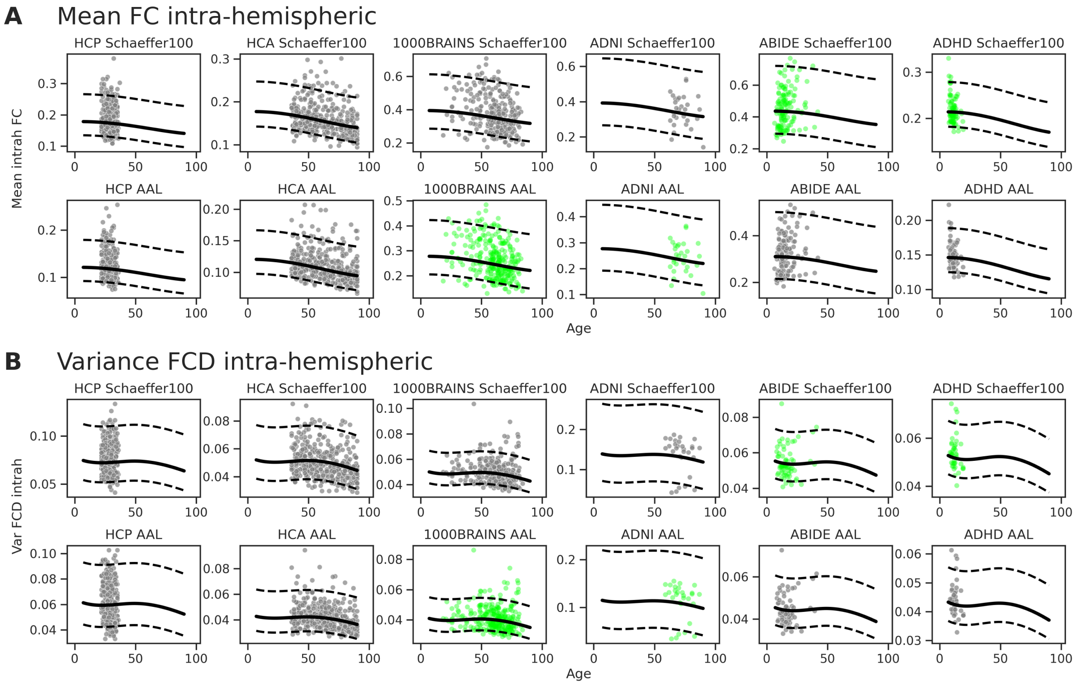


**Figure S1.** Cohort-specific normative trajectories and cross-atlas projections for (**A**) FC intra-hemispheric and (**B**) FCD intra-hemispheric. Solid lines and dotted boundaries represent the cohort- and atlas-specific median regression lines and normative quantiles estimated by the hierarchical GAMLSS model. Test data is shown in grey; the cross-atlas projections (in green) of test data for missing cohort-atlas combinations are generated by leveraging the model's estimated random effects.

bioRxiv preprint doi: https://doi.org/10.64898/2026.04.03.716292; this version posted April 6, 2026. The copyright holder for this preprint (which was not certified by peer review) is the author/funder, who has granted bioRxiv a license to display the preprint in perpetuity. It is made available under aCC-BY 4.0 International license.

| Metric | Top Performing Model(s) | ELPD | ELPD$_p$ | SE | $\Delta$ELPD | SE($\Delta$) |
|---|---|---|---|---|---|---|
| FC Interhem (Pearson) | SHASH$(\mu_L + b_0 + b_1x, \sigma + b_0, \nu, \tau)$ | 9063.8 | 26.2 | 74.6 | 0.0 | 0.0 |
| | SHASH$(\mu_S + b_0, \sigma + b_0, \nu, \tau)$ | 8967.8 | 46.9 | 76.1 | 96.0 | 104.9 |
| | **SHASH**$(\mu_L + b_0, \sigma + b_0, \nu, \tau)$ | 8954.9 | 25.5 | 74.7 | 108.9 | 104.5 |
| | SHASH$(\mu_S + b_0 + b_1x, \sigma + b_0, \nu, \tau)$ | 8952.9 | 50.1 | 75.4 | 110.8 | 105.4 |
| | $N(\mu_S + b_0, \sigma + b_0)$ | 8609.4 | 21.6 | 65.0 | 454.3 | 99.4 |
| | $N(\mu_L + b_0, \sigma + b_0)$ | 8492.4 | 48.2 | 68.5 | 571.3 | 101.2 |
| FC Intrahem (Pearson) | **SHASH**$(\mu_L + b_0, \sigma + b_0, \nu, \tau)$ | 9051.8 | 27.5 | 72.3 | 0.0 | 0.0 |
| | SHASH$(\mu_L + b_0, \sigma + b_0, \nu, \tau + b_0)$ | 8975.7 | 30.3 | 72.0 | 76.1 | 102.4 |
| | SHASH$(\mu_S + b_0 + b_1x, \sigma + b_0, \nu, \tau)$ | 8969.4 | 45.6 | 71.4 | 82.4 | 102.0 |
| | SHASH$(\mu_L + b_0 + b_1x, \sigma + b_0, \nu, \tau)$ | 8933.5 | 29.1 | 72.2 | 118.4 | 101.7 |
| | SHASH$(\mu_L + b_0, \sigma_L + b_0, \nu, \tau)$ | 8871.1 | 32.9 | 73.6 | 180.7 | 103.8 |
| FC Interhem (Spearman) | **SHASH**$(\mu_L + b_0, \sigma + b_0, \nu, \tau)$ | 9271.2 | 25.4 | 73.0 | 0.0 | 0.0 |
| | SHASH$(\mu_L + b_0, \sigma + b_0, \nu, \tau + b_0)$ | 9132.3 | 34.1 | 76.4 | 138.9 | 106.0 |
| | SHASH$(\mu_S + b_0, \sigma + b_0, \nu, \tau)$ | 9095.4 | 47.1 | 76.1 | 175.8 | 105.3 |
| | SHASH$(\mu_S + b_0 + b_1x, \sigma + b_0, \nu, \tau)$ | 9091.0 | 47.0 | 75.6 | 180.2 | 105.0 |
| | $N(\mu_L + b_0, \sigma_L + b_0)$ | 8748.6 | 31.3 | 69.0 | 522.6 | 100.2 |
| | $N(\mu_L + b_0, \sigma + b_0)$ | 8658.7 | 25.1 | 67.8 | 612.5 | 99.7 |
| | $N(\mu_S + b_0, \sigma + b_0)$ | 8625.7 | 44.6 | 67.3 | 645.5 | 98.3 |
| FC Interhem (Mutual Info) | **SHASH**$(\mu_L + b_0, \sigma + b_0, \nu, \tau)$ | 16642.9 | 27.0 | 81.9 | 0.0 | 0.0 |
| | SHASH$(\mu_L + b_0 + b_1x, \sigma + b_0, \nu, \tau)$ | 16557.7 | 33.3 | 85.0 | 85.2 | 118.6 |
| | SHASH$(\mu_S + b_0 + b_1x, \sigma + b_0, \nu, \tau)$ | 16407.3 | 61.7 | 86.5 | 235.6 | 118.6 |
| | SHASH$(\mu_L + b_0, \sigma_L + b_0, \nu, \tau)$ | 16399.6 | 42.1 | 88.4 | 243.3 | 120.2 |
| | $N(\mu_L + b_0, \sigma_L + b_0)$ | 16021.4 | 32.9 | 79.4 | 621.5 | 114.2 |
| | $N(\mu_L + b_0, \sigma + b_0)$ | 15833.0 | 26.2 | 81.3 | 809.9 | 114.3 |
| | $N(\mu_S + b_0, \sigma + b_0)$ | 15729.1 | 55.0 | 80.5 | 913.8 | 115.0 |
| FC Interhem (Coherence) | **SHASH**$(\mu_L + b_0, \sigma + b_0, \nu, \tau)$ | 10170.4 | 24.6 | 72.6 | 0.0 | 0.0 |
| | SHASH$(\mu_L + b_0, \sigma_L + b_0, \nu, \tau)$ | 10142.2 | 33.2 | 76.4 | 28.2 | 104.6 |
| | SHASH$(\mu_L + b_0 + b_1x, \sigma + b_0, \nu, \tau)$ | 9977.0 | 24.5 | 73.9 | 193.3 | 103.7 |
| | $N(\mu_L + b_0, \sigma + b_0)$ | 9457.1 | 23.7 | 69.5 | 713.3 | 100.4 |
| FC Interhem (Precision) | SHASH$(\mu_L + b_0, \sigma_L + b_0, \nu, \tau)$ | 20976.1 | 118.6 | 80.4 | 0.0 | 0.0 |
| | **SHASH**$(\mu_L + b_0, \sigma + b_0, \nu, \tau)$ | 20905.4 | 111.5 | 79.8 | 70.7 | 113.6 |
| | SHASH$(\mu_L + b_0 + b_1x, \sigma + b_0, \nu, \tau)$ | 20853.0 | 123.0 | 80.3 | 123.1 | 112.9 |
| | $N(\mu_L + b_0, \sigma + b_0)$ | 20589.0 | 61.3 | 81.2 | 387.2 | 114.5 |
| FCD Var Interhem | **SHASH**$(\mu_L + b_0, \sigma + b_0, \nu, \tau)$ | 14128.1 | 29.4 | 67.3 | 0.0 | 0.0 |
| | SHASH$(\mu_L + b_0, \sigma_L + b_0, \nu, \tau)$ | 14086.8 | 39.2 | 67.1 | 41.3 | 95.5 |
| | SHASH$(\mu_L + b_0 + b_1x, \sigma + b_0, \nu, \tau)$ | 14031.5 | 31.9 | 67.6 | 96.6 | 95.4 |
| | SHASH$(\mu_S + b_0, \sigma + b_0, \nu, \tau)$ | 13994.9 | 49.7 | 68.2 | 133.2 | 94.2 |
| | $N(\mu_S + b_0, \sigma + b_0)$ | 13576.3 | 46.9 | 54.5 | 551.7 | 86.5 |
| | $N(\mu_L + b_0, \sigma_L + b_0)$ | 13487.0 | 34.0 | 56.1 | 641.0 | 88.5 |
| FCD Var Intrahem | SHASH$(\mu_L + b_0, \sigma_L + b_0, \nu, \tau)$ | 13937.5 | 34.4 | 61.9 | 0.0 | 0.0 |
| | SHASH$(\mu_S + b_0 + b_1x, \sigma + b_0, \nu, \tau)$ | 13918.2 | 48.9 | 62.0 | 19.3 | 88.3 |
| | **SHASH**$(\mu_L + b_0, \sigma + b_0, \nu, \tau)$ | 13905.2 | 28.3 | 62.2 | 32.3 | 86.7 |
| | SHASH$(\mu_S + b_0, \sigma + b_0, \nu, \tau)$ | 13840.3 | 51.3 | 63.6 | 97.2 | 88.6 |
| | SHASH$(\mu_L + b_0 + b_1x)$ | -6274.1 | 0.0 | 48.7 | 20211.6 | 78.4 |

**Table S1.** Best statistical model specifications ranked by predictive accuracy: Expected Log Pointwise Predictive Density **(ELPD)**, Estimated effective number of parameters (**ELPD**$_p$), Standard Error of the ELPD (**SE**), Difference of ELPD from best model (**$\Delta$**ELPD) and Standard Error of the difference (SE(**$\Delta$**)).Models within 2 standard errors SE(**$\Delta$**) of the top-performing model are in black, other in gray. Only models that did not encounter warnings were reported*. The selected common reference model is shown in bold. Legend: $\mu_L$ = Linear trend; $\mu_S$ = Spline trend; $\sigma_L$ = Linear trend on scale; $b_0$ = Parameter-specific random intercept; $b_1x$ = Parameter-specific random slope. * Except for FC Interhem (Precision) where all the models had warnings.

bioRxiv preprint doi: https://doi.org/10.64898/2026.04.03.716292; this version posted April 6, 2026. The copyright holder for this preprint (which was not certified by peer review) is the author/funder, who has granted bioRxiv a license to display the preprint in perpetuity. It is made available under aCC-BY 4.0 International license.

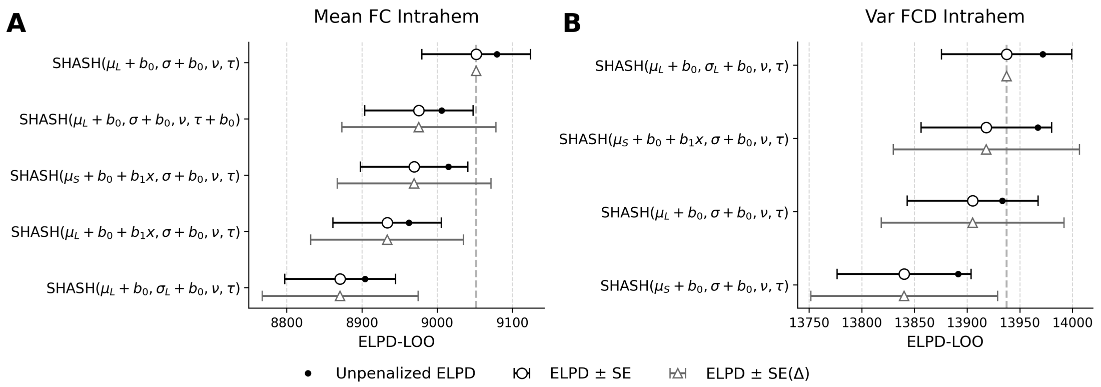


**Figure S2.** Bayesian model comparison for intrahemispheric functional connectivity (**A**) and fluidity via variance of dynamic functional connectivity (**B**). Models are ranked on the y-axis according to their predictive accuracy, with the best-performing (reference) model placed at the top. The x-axis represents the Leave-One-Out Expected Log Pointwise Predictive Density (ELPD-LOO), as the measure of predictive accuracy using cross-validation. The vertical dashed grey line marks the ELPD of the reference model. Black circles indicate the absolute ELPD estimate for each model, with horizontal black error bars representing $\pm 1$ standard error (SE) of that estimate. Grey triangles are plotted such that their visual distance from the vertical dashed line corresponds exactly to the difference in ELPD ($\Delta$ ELPD) compared to the top model. The horizontal grey error bars denote $\pm 1$ standard error of this difference, $SE(\Delta)$. We considers models ranking within $2 \times SE(\Delta)$ of the top model as statistically indistinguishable from it. Legend: $\mu_L$ = Linear trend; $\mu_S$ = Spline trend; $\sigma_L$ = Linear trend on scale; $b_0$ = Parameter-specific random intercept; $b_1 x$ = Parameter-specific random slope.

| | **FC Inter-hem (%)** | | **FC Intra-hem (%)** | | **FCD Inter-hem (%)** | | **FCD Intra-hem (%)** | |
|---|---|---|---|---|---|---|---|---|
| **Cohort** | **Control** | **Case** | **Control** | **Case** | **Control** | **Case** | **Control** | **Case** |
| HCP Young Adult | 77/636 (12.1%) | – | 76/636 (11.9%) | – | 56/633 (8.8%) | – | 57/633 (9.0%) | – |
| HCP Aging (HCA) | 89/748 (11.9%) | – | 85/748 (11.4%) | – | 60/748 (8.0%) | – | 58/748 (7.8%) | – |
| 1000BRAINS | 36/279 (12.9%) | – | 37/279 (13.3%) | – | 43/279 (15.4%)* | – | 39/279 (14.0%) | – |
| CAMCAN | 16/196 (8.2%) | – | 16/196 (8.2%) | – | 15/196 (7.7%) | – | 14/196 (7.1%) | – |
| ADNI | 2/29 (6.9%) | AD: 3/55 (5.4%)<br>MCI: 10/91 (11%) | 2/29 (6.9%) | AD: 2/55 (3.6%)<br>MCI: 11/91 (12.1%) | 3/29 (10.3%) | AD: 6/61 (9.8%)<br>MCI: 13/91 (14.3%) | 5/29 (17.2%) | AD: 7/61 (11.5%)<br>MCI: 18/91 (19.8%) |
| ABIDE | 12/132 (9.0%) | 35/374 (9.6%) | 9/132 (6.8%) | 32/374 (8.5%) | 10/65 (15.4%) | 24/195 (12.3%) | 7/65 (10.8%) | 29/195 (14.9%) |
| ADHD-200 | 4/84 (4.8%) | 19/215 (8.8%) | 7/84 (8.3%) | 23/215 (10.7%) | 5/42 (11.9%) | 10/102 (9.8%) | 5/42 (11.9%) | 17/102 (16.7%) |

**Table S2.** Percentage of extreme deviations ($|Z| > 1.645$) across healthy test controls and clinical cases for static and dynamic functional connectivity (denoted by FC and FCD, respectively). Values are presented as $n/N$ (%), where $n$ is the number of extreme deviations and $N$ is the total group size. To evaluate baseline calibration, local control groups were tested against the theoretical 10% expectation using exact Binomial tests (* $p_{FDR} < 0.05$). To evaluate clinical sensitivity, diagnostic groups were compared to their local, within-site control counterparts using Fisher's Exact Tests. After correcting for multiple comparisons, no diagnostic group exhibited a statistically significant inflation in extreme deviations relative to local controls (all $p_{FDR} > 0.05$).

bioRxiv preprint doi: https://doi.org/10.64898/2026.04.03.716292; this version posted April 6, 2026. The copyright holder for this preprint (which was not certified by peer review) is the author/funder, who has granted bioRxiv a license to display the preprint in perpetuity. It is made available under aCC-BY 4.0 International license.

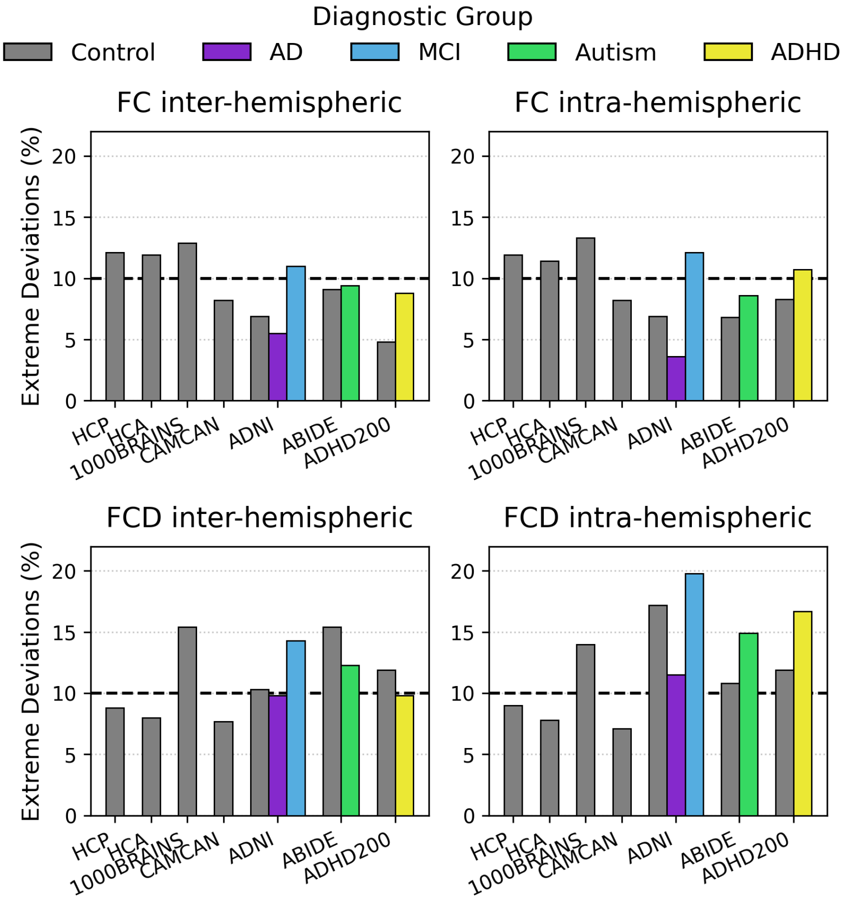


**Figure S3.** Percentage of extreme deviations ($|Z| > 1.645$) across healthy controls and clinical cases for static and dynamic functional connectivity (denoted by FC and FCD, respectively). The theoretical baseline is 10% (dashed black line).
